# Knee Flexion and Daily Activities in Patients following Total Knee Replacement: A Comparison with ISO Standard 14243

**DOI:** 10.1155/2015/157541

**Published:** 2015-08-11

**Authors:** Markus A. Wimmer, William Nechtow, Thorsten Schwenke, Kirsten C. Moisio

**Affiliations:** ^1^Department of Orthopedic Surgery, Rush University Medical Center, Chicago, IL 60612, USA; ^2^Feinberg School of Medicine, Northwestern University, Chicago, IL 60610, USA

## Abstract

Walking is only one of many daily activities performed by patients following total knee replacement (TKR). The purpose of this study was to examine the hypotheses (a) that subject activity characteristics are correlated with knee flexion range of motion (ROM) and (b) that there is a significant difference between the subject's flexion/extension excursion throughout the day and the ISO specified input for knee wear testing. In order to characterize activity, the number of walking and stair stepping cycles, the time spent with dynamic and stationary activities, the number of activity sequences, and the knee flexion/extension excursion of 32 TKR subjects were collected during daily activity. Flexion/extension profiles were compared with the ISO 14243 simulator input profile using a level crossing classification algorithm. Subjects took an average of 3102 (range: 343–5857) walking cycles including 65 (range: 0–319) stair stepping cycles. Active and passive ROMs were positively correlated with stair walking time, stair step counts, and stair walking sequences. Simulated knee motion according to ISO showed significantly fewer level crossings at the flexion angles 20–40° and beyond 50° than those measured with the monitor. This suggests that implant wear testing protocols should contain more cycles and a variety of activities requiring higher knee flexion angles with incorporated resting/transition periods to account for the many activity sequences.

## 1. Introduction

Total knee replacement (TKR) surgery has become the most common total arthroplastic procedure in the United States with over 650,000 surgeries being performed in 2010 and rising to an expected 1.4 million annual surgeries by 2020 [1]. In addition, TKR surgeries are increasingly performed on younger and more active patients [2]. In this patient group, polyethylene wear can be a limiting factor for longevity [3]. As for the hip, wear particles generated during sliding contribute to osteolysis and subsequent loosening of the prosthesis [4]. Since this is one of the most common reasons for revision in TKR [5], preclinical wear testing is an important step before any new TKR device is brought to market.

State-of-the-art knee wear testing is conducted according to ISO standards 14243-1 [6] and/or 14243-3 [7]. These standardized protocols presumably mimic the* in vivo* kinematic and kinetic conditions of the knee prosthesis during its lifetime. The input for TKR wear testing is specified as a sequence of gait cycles that are continuously repeated at 1.0 ± 0.1 Hz until 5 million cycles are reached. This, as commonly assumed, represents the prosthetic life span of about five years* in vivo*. Indeed, several activity studies on patients with total hip and/or knee replacement found that subjects walk on average between 0.9 and 1.4 million gait cycles annually [8–10].

However, walking is only one of many daily activities performed by patients following TKR. Other common activities include standstill with related starting/stopping maneuvers, stair ascent/descent, chair sitting and rising, lying down to rest, and a variety of recreational activities. Hence, including kinematic and kinetic characteristics of these activities into wear testing may result in a more realistic simulation of wear. Indeed, better agreement between wear patterns on simulator tested prostheses and those observed on retrieved specimens was achieved after incorporating stair descent into testing protocols [11, 12]. However, for TKR subjects, duration and frequency of these additional activities are unknown. Therefore, the purpose of the study was to describe the frequency and duration of daily physical activities of TKR subjects during a 12-hour day using electrogoniometry. In addition, we chose to follow the flexion/extension excursion of the knee prosthesis throughout the day, because flexion/extension movement is an input variable for the knee simulator, which directly impacts sliding distance and, thus, wear. The flexion/extension excursion is also interesting from a clinical viewpoint: Active and passive knee flexion ROM are indicators of a patient's functional status, and knee ROM is commonly used to evaluate TKR surgery [13] and rehabilitation programs [14]. Although, TKR ROM has been found uncorrelated with patient satisfaction and perceived improvement in quality of life [15], it is unknown if TKR ROM is associated with activity profile. We hypothesized that (a) subject activity characteristics are correlated with knee flexion range of motion (ROM) and (b) there is a significant difference between subject flexion/extension excursion movement and the ISO 14243 simulator input.

## 2. Subjects and Methodology

### 2.1. Subject Population

Forty subjects were recruited from a large orthopedic practice (Midwest Orthopaedics, Chicago, IL) specialized in joint replacement surgery. The study was approved by the institutional review board, and all subjects gave informed consent. Potential subjects were identified from a database of all patients who had received a TKR at the Medical Center. All participants met the following inclusion criteria: having received a primary TKR implant of a single design (Miller-Galante or MGII, Zimmer Inc., Warsaw, IN, USA), having knee in excellent condition as determined by latest follow-up, being able to walk without assistive devices, and being able to live and function independently in their home. Exclusion criteria were as follows: past or present history of a neurologic disorder; other medical conditions affecting their physical function; previous revision surgery. Six subjects were excluded from the analysis because of cable or connector failure of the electronic data recording device and two subjects were excluded because of other technical errors which truncated the activity data. Data for the remaining 32 subjects were included in the analysis (Table 1).

### 2.2. Activity Monitor

**Figure 1:**
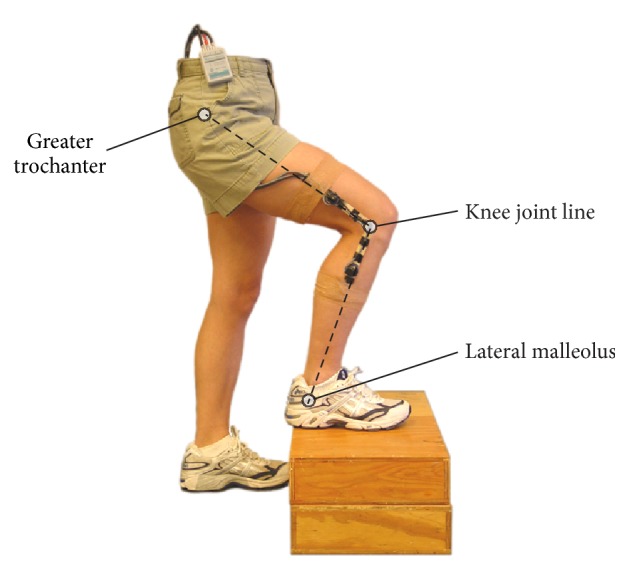
Placement of the activity monitor. The following anatomical landmarks served as orientation: greater trochanter, knee joint line, and lateral malleolus. The electrogoniometer was placed on the lateral aspect of the knee joint line. The two monitor segments were aligned along lines connecting the landmarks.

The activity monitor utilized hardware introduced by Morlock et al. [9] and a portable data logger collecting data from three sensors at 30 Hz. Two inclination sensors recorded sagittal plane thigh and shank inclinations. A goniometer connecting the two device segments measured the knee flexion angle (Figure 1). The device weighed less than 100 g and did not inhibit movement. Normal clothing was worn over the device.

**Figure 2:**
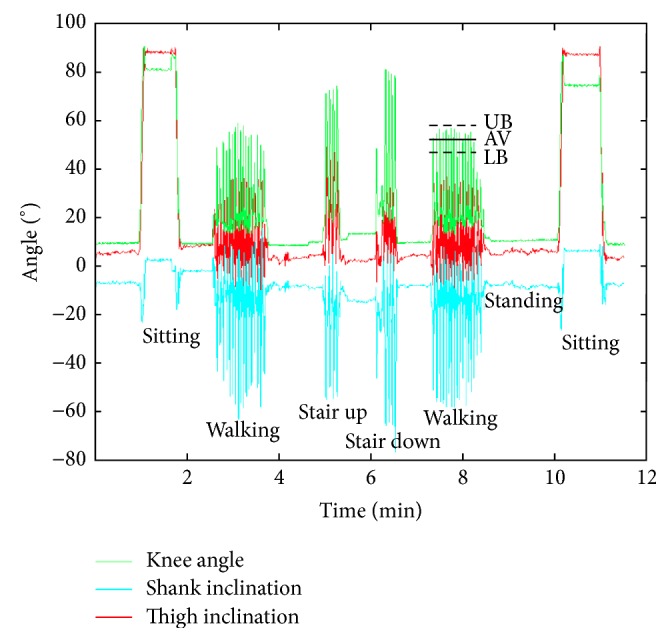
Thigh and shank inclination angles, as well as knee flexion angle for various activities of a representative subject during the calibration procedure. Zero-degree flexion and zero-degree inclination indicate a straight knee and vertical limbs (e.g., during standing). LB = lower boundary, UP = upper boundary, and AV = average.

Data was streamed to a memory card embedded in the data logger. The postprocessing code was written in MATLAB (MathWorks, Inc., Natick, MA, USA). Dynamic activities were classified into walking, stair stepping (ascending and descending combined), and unrecognized activities based on a pattern recognition program previously written [9] and adapted for TKR by Hänni et al. [16]. Lower and upper flexion angle boundaries for activity recognition were manually set for each subject using data captured during a calibration run (Figure 2). Stationary activity, for example, lying down, sitting, and standing, was identified as a period with the thigh and shank inclination sensors remaining within a ±4° range for at least 1.2 seconds and was further classified based on limb inclination (Table 2).

Output of the analysis software included the number of sequences for each activity, the time of each sequence, the overall time for each activity, and the number of cycles for level and stair walking. A sequence was defined as a continuous activity within its respective boundary conditions. All data were normalized to 12 hours to allow for comparison between subjects.

### 2.3. Monitor Validation

Twenty out of 32 subjects were filmed for approximately two minutes (2.3 ± 0.8 minutes) while performing sequences of sitting, standing, lying down, walking, and ascending and descending stairs (simultaneous to activity monitor recording). Four subjects were filmed for 53–95 minutes while performing routine daily activities. Two blinded observers, who did not otherwise participate in the study, independently watched the videos. The number of cycles walked or climbed was counted; times spent with lying down, sitting, standing, walking, and stair stepping were measured; stationary, dynamic, and total activity times were calculated. Since the intraclass correlation coefficient (ICC) between the two observers ranged from 0.86 for lying down time to values greater than 0.99 for stair stepping, for both the short and long videos, the observers' measurements were subsequently averaged. The observer-averaged data were then used for comparison with the monitor-derived data.

No systematic offset between video and activity monitor measurements was detected. For the short videos, ICCs for all parameters, with the exception of sitting time (ICC = 0.60), exceeded 0.8 (range: 0.80 to 0.98). For the long videos, ICCs exceeded 0.9 for all parameters (Table 3). The high ICC for sitting time measured from the longer videos (ICC = 0.98) confirmed the monitor's utility to track this activity in the field.

### 2.4. Testing Procedure

During a brief clinical examination of the subjects (at their home) by a licensed physical therapist, height and weight, as well as active and passive knee flexion range of motion (ROM), were measured. Double sided Velcro tape (Velcro Inc., Manchester, NH, USA) and Elastikon athletic tape (Johnson & Johnson Inc., New Brunswick, NJ, USA) were used to attach the activity monitor to the skin of the subjects. An elastic tube stocking was pulled over the affected leg to prevent chafing of the device against cloths and to protect the cables from entanglement. Prior to data collection, each subject performed an activity calibration protocol consisting of sitting, standing, level walking, and stair walking, during which the subject was filmed and sensor data were recorded. Subsequently, the activity monitor was restarted to begin the actual data collection. The calibration procedure was repeated before the monitor was detached at the end of data collection to detect potential sensor shifting or other changes. Subjects were asked to keep a diary of their activities and to follow their usual activity patterns throughout the day. Data collection was initialized as early as 30 minutes of the subject's waking time and ended as late as bed time to capture data for approximately 12 hours.

### 2.5. Comparison of TKR Flexion/Extension Excursions with ISO Simulator Profile

**Figure 3:**
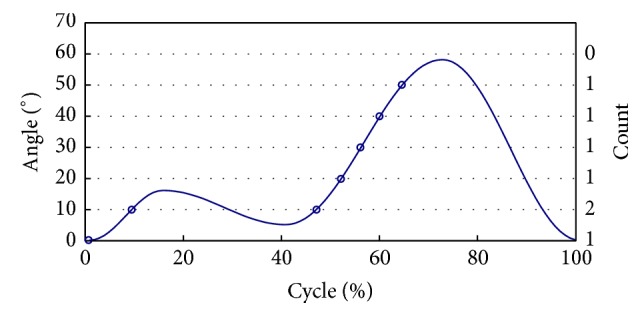
Level crossing classification of the flexion angle *α* during one typical walking cycle (duration: about 1 s). The count of each level is summarized to the right.

The TKR flexion/extension curves from the subjects were compared to the flexion/extension curve specified in the standard ISO standard by using the concept of “level crossings.” Referring to a graph of knee flexion (*y*-axis) versus percent gait cycle (*x*-axis), a level crossing is a point where the flexion/extension curve crosses the horizontal line denoting a specified knee angle level (Figure 3). As the flexion/extension curve goes up and down, there can be zero to multiple such crossings for each angle level. The number of level crossings for the ISO flexion/extension curve and for the flexion/extension curve of each subject was counted at the 0, 10, 20, …, 140° angle levels. Only upward crossings were counted (Figure 3). Assuming an identical number of walking cycles per day, the ISO-simulated knee flexion/extension level crossings were now compared to those of the TKR subjects.

### 2.6. Statistical Analysis

All statistical tests were performed in SPSS Version 16.0 (SPSS Inc., Chicago, IL, USA). After normalization to 12 hours, the mean and standard deviations were computed for the relative amount of time spent with each activity, the occurring sequences for each activity, and the number of steps for level walking and stair walking. Linear regression models were used to identify associations between these monitor-derived values and subject characteristics including time past surgery, BMI, height, mass, age, and active and passive ROM. One-sample *t*-tests were used to detect significant differences in number of level crossings between the* in vivo* activity data and the value for the ISO standard at each angular level. The significance level for all statistical tests was set to 0.05. The Bonferroni correction was applied for tests with multiple comparisons.

## 3. Results

The average total test duration was 11.3 ± 1.2 hours (range: 8.1–13.0 hours) out of which 9.3 ± 1.2 hours were identified as stationary activities and 0.9 ± 0.5 hours consisted of dynamic activities. The remainder of 1.1 ± 0.4 hours could not be allocated by the analysis software and was marked “unrecognized.” The most frequently performed activity, according to sequence counts, was standing, followed by level walking, sitting, stair walking, and lying down (Table 4). Subjects performed an average of 3102 ± 1553 walking cycles per 12 hours of daily activity, out of which 65 ± 70 were stair cycles (2.1%) (Table 4). The number of walking cycles correlated with the number of walking sequences (*r* = 0.743; *P* < 0.001). On average, subjects took 8.3 ± 3.0 walking cycles per walking sequence. Subjects spent significantly more time sitting than performing any other activity (Table 4; *P* < 0.001). Subjects spent significantly less time walking than standing (Table 4; *P* < 0.001).

Active knee flexion ROM (as measured during the clinical exam) correlated with stair walking time (*r* = 0.532, *P* = 0.002), stair walking counts (*r* = 0.551, *P* = 0.001), and stair walking sequences (*r* = 0.556, *P* = 0.001). Similarly, passive knee flexion ROM correlated with stair walking time (*r* = 0.534, *P* = 0.002), stair walking counts (*r* = 0.535, *P* = 0.002), and stair walking sequences (*r* = 0.538, *P* = 0.001). Time between surgery and activity analysis did not correlate with any of the functional variables. No statistically significant difference between female and male subjects for any of the variables was found, except for height (*P* < 0.001).

**Figure 4:**
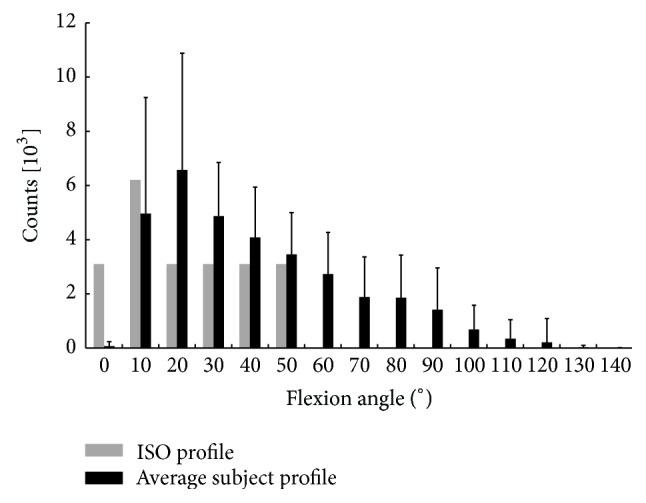
Mean (1 SD) number of level crossings for flexion angle levels from 0 to 140°. Standard deviations are indicated for the average counts of the activity analysis patient population. The ISO profile count was extrapolated from the flexion-extension curve as provided by ISO 14243 (ISO-14243-1, 2002) and the average number of walking steps taken by the subjects during a 12-hour period.

The level crossing classification indicated that the population as a whole crossed flexion levels ranging from 0° to 140° approximating a log-normal distribution (Figure 4). The 20° flexion level was crossed most frequently with an average of 6789 ± 4376 crossings. The 140° level was crossed the least, averaging only 2 ± 11 level crossings in the day. However, not all TKR subjects crossed all levels during daily activity. The 0° level was crossed by 20 subjects (although only by six at a relevant number of >100), and the 140° level was crossed by only three subjects. All TKR subjects crossed levels between 10° and 70°. There was a significant correlation between the subjects' maximum level crossed and the measured active or passive ROM (*P* < 0.001).

The range of crossed levels for ISO was much smaller (0° to 50°) following a nonnormal distribution. Comparing it to subject data, the level crossing pattern was shifted to the left (i.e., towards lower flexion angles; see Figure 4). Most level crossings were found for the 10° angle (instead of the 20° angle). Beyond the 50° angle there were no crossings at all. The average number of crossings was higher for the subject population at all flexion angles beyond 10°. This finding was significant (*P* < 0.01), except for the 50° angle (Figure 4).

## 4. Discussion

This study provides information on the frequency and duration of daily physical activities performed by TKR patients during a 12-hour day. Subjects spent most of the time sitting, followed by standing and walking. The large number of activity sequences (mean total number of sequences: 2489) indicates that common daily activities such as standing are interspersed with frequent transitions between activities resulting in ever changing* in vivo* loading profiles for the implant. More distinct sequences of standing were recorded than of any other activity. The results suggest that standing is a common resting state between various dynamic activities. In simulation experiments of total hip joints, resting periods increased the starting friction, indicating lubricant starvation, potentially leading to increased wear [17]. The results of this study suggest that one resting period should theoretically be included on the simulator every 8.2 cycles to properly reflect the dynamic activity profile of walking. Subjects who took more walking cycles did so during a greater number of sequences, and the number of walking cycles per sequence showed a relatively small variability. These results suggest that wear profiles of more active patients could be simulated by longer testing times.

The total numbers of walking cycles taken per day in this subject population are within the ranges reported in the literature. A recent meta-analysis by Naal and Impellizzeri [10], which included 2460 patients with total joint replacement (summarizing data from 26 pedometer/accelerometer studies), found a weighted mean of 3360 (95% CI: 2872–3849) walking cycles per day. This compares well with our own average of 3102 walking cycles per day, particularly if the somewhat older age of our subject population is taken into account. The number also agrees with another meta-analysis of healthy individuals: Bohannon [18] found 3250 walking cycles in individuals over 65 years old. Since our TKR patients are expected to take 1.13 ± 0.56 million walking cycles per year, including about 23,700 stair stepping cycles, they are however more active than what is normally assumed in wear simulations. In general, a large variability in activity and step patterns was observed between subjects. The most active patient is estimated to take 2.33 million walking cycles per year, including 116,000 stair stepping cycles. Similar results have been reported for patients following total hip arthroplasty [9]. The large variability in the number of waking cycles per day suggests that results from wear tests are only representative for some subjects and that larger total numbers of cycles per wear test are needed to simulate wear patterns for more active patients.

Flexion ROM is an important outcome variable in TKR since many daily activities depend on it. As has been recently summarized by Fu et al. [19], a higher ROM than walking is necessary for stair or chair maneuvers (90°–120°), kneeling or squatting (110°–165°), bathtub use (135°), and gardening (>150°). Not surprisingly, in this study, there was a high correlation between the maximum flexion angle measured during daily activity and the ROM measured during clinical examination. Interestingly, subjects with greater active and passive knee flexion ROM also spent more time level walking and stair stepping. However, it is unclear if more active patients had greater knee flexion ROM because they were more active or if greater knee flexion ROM facilitated a greater activity level. Nevertheless, the association between knee flexion ROM and activity level should be taken into consideration during rehabilitation programs following TKR surgery. The findings are also interesting in the context of the ongoing debate about the usefulness of high-flexion knee implants [19, 20]. Based on this data, active patients might very well benefit from it. Future studies comparing high-flexion and standard TKR should therefore stratify for activity level to break the stalemate.

The level crossing analysis for activities during a 12-hour period revealed a large range of knee flexion during daily activities. The most frequently crossed angle was 20° of knee flexion in our subject population and some subjects flexed their prosthetic knee up to 140°. In contrast, the most frequently crossed angle as specified in the ISO 14143 standard was 10° of knee flexion with a maximum knee flexion angle crossing at 50°. While it is well known that the ISO standard is representative for walking activities, the results of this study clearly show that the ranges of knee flexion experienced* in vivo* are not fully represented by the ISO profile. Hence, the ASTM F04 committee has become active in the development of a standard guide, which will include loading profiles other than walking (personal communication). Since the medial and lateral femoral radii of the TKR typically decrease with higher flexion angle, stresses at the polyethylene plateau may increase leading to more surface damage. These differences may explain the discrepancies between wear patterns on retrieval prostheses and those on simulator wear tested prostheses [12, 21]. Hence, a modified simulator input profile entailing the flexion profile of activities other than walking is necessary to simulate* in vivo* loading and wear of the implant.

Recently, detailed* in vivo* loading data for daily activities in patients following TKR has become available [22–24]. While these studies specified the* in vivo* load magnitude and knee flexion angles for different activities of daily living, the data in these studies were captured from a relatively small patient pool with instrumented knee implants and usually collected in a laboratory environment, except for D'Lima et al. [23] who conducted some field measurements for specific activities. However, combining the contact force information reported in the literature with the activity profiles obtained in this study greatly improves the understanding of* in vivo* loading profiles during daily activities in patients following TKR. Based on the results of the present study, a ratio of 47 : 1 of number of walking cycles to the number of stair stepping cycles would be appropriate to represent loading patterns during locomotion of daily living.

The study has several limitations. All subjects in this study had received a Miller-Galante or MGII implant. It is possible that activity profiles differ between implant type and model, that they change over time, and that these changes may affect implant wear patterns. Also, the advanced age of the subject population (mean: 77.8 years) may have affected the activity pattern; however, as discussed above, the observed number of walking cycles was well within the range reported in the literature. Thus, we believe this should be similarly true for other outcome variables of this study.

The amount of unrecognized activity (11.7% of the total measurement time) was unexpectedly high. A detailed analysis of the recorded waveforms of several subjects revealed that this unrecognized data set consisted mostly of transitions from one activity to another. Explicit definitions for transitions between activities would improve the proper allocation of time. Further, some patients walked with two distinguishable types of step patterns: normal walking steps with a high flexion angle and so-called “fine steps” characterized by lower knee flexion angle. Fine steps with a peak flexion angle below the lower boundary of level walking were not recognized and classified as “unrecognized.” These fine steps were often taken in confined spaces such as the kitchen as indicated by the patients' diaries. Future refinements of the recognition algorithm should incorporate these additional distinctions of dynamic activities. Finally, activity and flexion/extension monitoring of the knee occurred without simultaneous recording of knee contact force, which comprises another important input variable for knee wear testing. Future studies are necessary to determine the specific loading profile occurring at flexion angles >60°.

## 5. Conclusion

In conclusion, walking and stair stepping accounted for about 10% of the monitoring time, with a ratio of 47 : 1. Subjects with a higher knee ROM climbed more stairs. While level walking is the dynamic activity that the artificial implant will have to endure the most, transition periods between activities are quite common. Walking sequences often include periods of standing. The knee flexion excursion during 12 hours of daily activity in patients following TKR includes knee flexion angles ranging from 60° to 130°, which is not represented by the current ISO standards. Taken together, simulated implant wear testing should contain resting or transition periods between activities and a larger range of activities such as stair walking and chair maneuvers and include more loading cycles than specified in the current standard.

## Figures and Tables

**Table 1:** Mean, one standard deviation, and range of demographic and functional data for subjects included in the analysis (*N* = 32).

Parameter	Mean (1 SD)	Range
Gender M/F	10/22	N/A
Age [years]	77.8 (6.1)	66.0–89.0
Height [cm]	164.6 (10.0)	149.5–186.0
Mass [kg]	80.8 (16.4)	57.4–117.1
BMI [kg/m^2^]	29.9 (5.7)	21.2–44.3
Affected and tested side (right/left)	14/18	N/A
Time between surgery and data collection [years]	11.5 (3.3)	6.6–21.9

Active knee ROM		
Flexion [°]	108 (12)	88–141
Extension [°]	2 (3)	−6–9
Passive knee ROM		
Flexion [°]	110 (13)	88–141
Extension [°]	2 (4)	−6–9

ROM: range of motion.

**Table 2:** Classification of stationary activities into lying down, sitting, and standing was based on shank and thigh inclination.

Activity	Shank inclination [°]	Thigh inclination [°]	Minimum duration [second]
Lying down	>85	>85	6
Sitting	>85	30–120	3
Standing	−20–20	−10–45	3

**Table 3:** Validation data for activity monitor results and observations of long videos for four subjects. Time values were rounded to the nearest minute (except for stair walking time). All intraclass correlation coefficients were statistically significant (*P* < 0.04).

Parameter	Activity	Monitor results	Observer results	ICC
Time [min]	Lying down	4 ± 1	4 ± 2	0.99
	Sitting	18 ± 4	18 ± 4	0.98
	Stair walking	0.73 ± 0.56	0.64 ± 0.38	0.93
	Level walking	21 ± 11	25 ± 11	0.99
	Standing	21 ± 11	24 ± 13	0.90
	Total stationary	44 ± 10	46 ± 12	0.91
	Total dynamic	22 ± 12	26 ± 11	0.92
	Overall total	66 ± 15	72 ± 19	0.91

Steps	Level walking	1121 ± 607	1148 ± 594	0.99
	Stair walking	34 ± 22	32 ± 20	0.99

**Table 4:** Mean, one standard deviation, and range of relative time, step counts, and number of sequences for all tested activities for all included subjects (*N* = 32). Time and steps were normalized to 12 hours.

Parameter	Activity	Mean (1 SD)	Range
Time [%]	Lying down	7.8 (10.5)	0.0–36.5
	Sitting	59.9 (14.7)	33.6–96.3
	Standing	12.7 (5.8)	0.6–25.8
	Level walking	7.8 (3.8)	1.1–14.5
	Stair walking	0.1 (0.1)	0.0–0.6
	Unrecognized	11.7 (3.6)	1.4–15.5

Steps	Stair walking	65 (70)	0–319
	Level walking	3037 (1523)	343–5857
	Total	3102 (1553)	343–6113
	Pedometer	2796 (1848)	258–7152

Sequence	Lying down	2 (3)	0–11
	Sitting	57 (31)	25–144
	Standing	514 (233)	25–945
	Level walking	357 (165)	57–752
	Stair walking	8 (14)	0–70
	Unrecognized	1551 (689)	184–3001
